# Complete Genome Sequence of *Veillonella atypica* OK5, the First Transformable Strain in the Species

**DOI:** 10.1128/genomeA.00391-17

**Published:** 2017-06-01

**Authors:** Peng Zhou, Gary Xie, Xiaoli Li, Jinman Liu, Fengxia Qi

**Affiliations:** a Department of Microbiology and Immunology, College of Medicine, University of Oklahoma Health Sciences Center, Oklahoma City, Oklahoma, USA; b Division of Oral Biology, College of Dentistry, University of Oklahoma Health Sciences Center, Oklahoma City, Oklahoma, USA; c Bioscience Division, Los Alamos National Laboratory, Los Alamos, New Mexico, USA

## Abstract

The *Veillonella atypica* strain OK5 was isolated from a human saliva sample and was the first strain shown to be genetically transformable in the *Veillonella* genus. Genetic studies using this strain have helped us gain much insight into the ecology of human oral biofilms. Here, we report the complete genome sequence of *V. atypica* OK5.

## GENOME ANNOUNCEMENT

Veillonellae are one of the most predominant bacteria commonly found in the plaques of the human oral cavity, and have been shown to coaggregate with a number of colonizers in all colonizing periods, thus making them vital in the oral biofilm ecology (1–4). *Veillonella atypica* OK5 was isolated from a human saliva sample and was the first transformable strain in the *Veillonella* genus (5, 6). Recently, a counterselectable markerless mutagenesis system was successfully established in this strain (7), making it a more robust model system in genetic studies of *Veillonella*. To further facilitate future studies using this strain, we sequenced the complete genome of the *V. atypica* OK5.

*V. atypica* OK5 was cultivated in anaerobic condition (85% N_2_, 10% CO_2_, and 5% H_2_), at 37°C, in brain heart infusion broth supplemented 0.6% sodium lactate. The fresh culture was harvested and lysed for 2 h in TE-buffer (pH 8.0) plus lysozyme (10 mg/mL). Genomic DNA was isolated using the Wizard genomic DNA purification kit (Promega). The genome was sequenced at the Laboratory for Molecular Biology and Cytometry Research at University of Oklahoma Health Sciences Center (OUHSC), using an Illumina MiSeq Next Generation sequencer, and generating 1,475,302 paired-end reads. The sequence reads were assembled *de novo* with CLC Genomics Workbench, which generated 136 contigs. Multiplex PCR was utilized to identify the relationship of the contigs (8). The gaps were filled by PCR product sequencing using ABI 3730XL capillary sequencer at the OUHSC core facility. The length of the genome is 2,071,952 bp, and the G+C content is 39.11%.

The genome sequence was annotated using the JGI IMG annotation pipeline (9). Annotation identified that the genome encodes 1,897 predicted proteins and 45 tRNAs and possesses 4 copies of 5S-16S-23S rRNA genes. Analyses against the Human Oral Microbiome Database (HOMD) (10) identified 8 putative hemagglutinin genes (*hag*) in OK5, indicating its bridging role in the formation of human oral biofilm community (2). Hag1 adhesin has been identified to be responsible for OK5 coaggregating with 3 streptococcal species (*Streptococcus gordonii*, *Streptococcus cristatus*, and *Streptococcus oralis*), *Porphyromonas gingivalis* and human buccal cells by mutagenesis of *hag1* gene (3). Analyses of the genome sequence with Kyoto Encyclopedia of Genes and Genomes (KEGG) (11) confirmed the presence of the complete pathways for the heme and vitamin K biosynthesis. We have reported that the heme biosynthesis pathway is not only functional in OK5, but required for facilitating the growth of periodontal pathogen (*P. gingivalis*) *in vitro* (12). Interestingly, a number of genes encoding putative transposase (13) were present in the OK5 genome, implying this strain possesses selective advantage in human oral cavity (14). Sequencing of the *V. atypica* OK5 genome shows it to be the only strain with both genetic system (6, 7) and genome information in the *Veillonella* genus, and might deepen our understanding of its bridging role in the formation of oral biofilm and the development of periodontal diseases.

### Accession number(s).

The complete genome sequence of *Veillonella atypica* OK5 has been deposited at GenBank under the GenBank accession no. CP020566.
